# A Text Messaging Intervention to Improve Heart Failure Self-Management After Hospital Discharge in a Largely African-American Population: Before-After Study

**DOI:** 10.2196/jmir.2317

**Published:** 2013-03-11

**Authors:** Shantanu Nundy, Rabia R Razi, Jonathan J Dick, Bryan Smith, Ainoa Mayo, Anne O'Connor, David O Meltzer

**Affiliations:** ^1^Section of General Internal MedicineDepartment of MedicineUniversity of Chicago Medical CenterChicago, ILUnited States; ^2^Cedars-Sinai Heart InstituteLos Angeles, CAUnited States; ^3^Department of MedicineColumbia University College of Physicians and SurgeonsNew York, NYUnited States; ^4^Department of MedicineUniversity of Chicago Medical CenterChicago, ILUnited States; ^5^Section of Hospital MedicineDepartment of MedicineUniversity of Chicago Medical CenterChicago, ILUnited States; ^6^Section of CardiologyDepartment of MedicineUniversity of Chicago Medical CenterChicago, ILUnited States

**Keywords:** heart failure, self-care, patient education, cellular phone, text messaging, African Americans

## Abstract

**Background:**

There is increasing interest in finding novel approaches to reduce health disparities in readmissions for acute decompensated heart failure (ADHF). Text messaging is a promising platform for improving chronic disease self-management in low-income populations, yet is largely unexplored in ADHF.

**Objective:**

The purpose of this pre-post study was to assess the feasibility and acceptability of a text message–based (SMS: short message service) intervention in a largely African American population with ADHF and explore its effects on self-management.

**Methods:**

Hospitalized patients with ADHF were enrolled in an automated text message–based heart failure program for 30 days following discharge. Messages provided self-care reminders and patient education on diet, symptom recognition, and health care navigation. Demographic and cell phone usage data were collected on enrollment, and an exit survey was administered on completion. The Self-Care of Heart Failure Index (SCHFI) was administered preintervention and postintervention and compared using sample *t* tests (composite) and Wilcoxon rank sum tests (individual). Clinical data were collected through chart abstraction.

**Results:**

Of 51 patients approached for recruitment, 27 agreed to participate and 15 were enrolled (14 African-American, 1 White). Barriers to enrollment included not owning a personal cell phone (n=12), failing the Mini-Mental exam (n=3), needing a proxy (n=2), hard of hearing (n=1), and refusal (n=3). Another 3 participants left the study for health reasons and 3 others had technology issues. A total of 6 patients (5 African-American, 1 White) completed the postintervention surveys. The mean age was 50 years (range 23-69) and over half had Medicaid or were uninsured (60%, 9/15). The mean ejection fraction for those with systolic dysfunction was 22%, and at least two-thirds had a prior hospitalization in the past year. Participants strongly agreed that the program was easy to use (83%), reduced pills missed (66%), and decreased salt intake (66%). Maintenance (mean composite score 49 to 78, *P*=.003) and management (57 to 86, *P*=.002) improved at 4 weeks, whereas confidence did not change (57 to 75, *P*=.11). Of the 6 SCHFI items that showed a statistically significant improvement, 5 were specifically targeted by the texting intervention.

**Conclusions:**

Over half of ADHF patients in an urban, largely African American community were eligible and interested in participating in a text messaging program following discharge. Access to mobile phones was a significant barrier that should be addressed in future interventions. Among the participants who completed the study, we observed a high rate of satisfaction and preliminary evidence of improvements in heart failure self-management.

## Introduction

Despite major scientific advances, heart failure continues to be a common and costly condition, and each year over 1 million people are admitted to an inpatient setting for acute decompensated heart failure (ADHF) [1,2]. National attention has turned toward reducing 30-day readmissions for ADHF, partially because financial penalties from the Centers for Medicare and Medicaid Services (CMS) for higher than expected rates of readmissions began in October 2012 [3,4]. This problem is particularly salient to hospitals serving larger proportions of African Americans because these patients have higher rates of readmissions than white patients [5-7]. Thus, there is an urgent need for low-cost solutions to reduce heart failure readmissions in African Americans.

Approximately 40% of ADHF hospitalizations are preventable because of varied factors, such as dietary indiscretion, medication nonadherence, and lack of timely medical consultation [8]. Patient nonadherence to heart failure drugs ranges from 30% to 60% and nonadherence to lifestyle recommendations ranges from 50% to 80%, with higher rates occurring in socioeconomically disadvantaged groups [9].

Mobile technology, in particular text messaging (also known as SMS: short message service), is emerging as a promising platform for chronic disease management in low-income populations [10,11], in part because it has high rates of utilization across socioeconomic groups [12,13]. Recent studies of mobile phone-based telemonitoring interventions in heart failure have demonstrated mixed success in reducing heart failure readmissions [14,15]. However, these interventions were not designed or evaluated in African American patients and may not be as effective in these populations. For one, these interventions typically required Internet- or Bluetooth-enabled phones, which may not always be available in these communities. Second, these interventions largely focused on telemonitoring rather than self-management support. The results of a recent large multicenter clinical trial found that self-management education was effective only in low-income patients (<$30,000 family income) [16], suggesting that a one-size-fits-all approach to improving heart failure outcomes does not work, and that self-management education is particularly effective in vulnerable health populations.

In this study, we pilot-tested a text message–based self-management intervention in an urban, largely African American population for 30 days following hospitalization for ADHF. Our study aims were to assess the feasibility and acceptability of the intervention and to test the hypothesis that the intervention was associated with improvements in self-management.

## Methods

### Patient Recruitment

After study approval was obtained from the Institutional Review Board, patients were recruited from the University of Chicago Medical Center (UCMC) inpatient cardiology service. Patients were recruited until target enrollment was achieved from November 2011 to January 2012 for a 4-week study. Informed consent was obtained prior to recruitment. Eligible patients included adult patients over the age of 18 years who were diagnosed with ADHF either with decreased or preserved systolic function as determined by the admitting physicians. Because the intent of the study was to provide self-management support, individuals who were not their own primary caregiver, were being discharged to a rehab facility, or who had poor mental status (Mini-Mental score<17) were excluded [17]. In addition, patients who did not have access to a personal mobile phone were not eligible for enrollment.

Consent was obtained from the attending physicians for contacting their patients for enrollment. Discharge planners were encouraged to notify the study team of any new admissions for ADHF. Study participants received $30 for study participation and to offset the costs of text messages. They were also provided with a scale to measure their weight.

### Study Design

The pilot was designed as a single-arm prospective study. The primary endpoint was change in the Self-Care of Heart Failure Index (SCHFI), a well-described measure of self-management in heart failure [18], which was administered at enrollment and at the end of the 30-day intervention. In addition, a mobile phone usage survey was administered on enrollment [19], and demographic and clinical data were obtained through chart review. At the completion of the intervention, a telephone-based patient experience survey, including Likert-scale and open-ended questions, was administered [19].

### Study Intervention

A text message communication platform developed for health researchers, SMS-Care (mHealth Solutions LLC, New York, NY, USA), was used for this study. Participants were enrolled in SMS-Care prior to discharge from the hospital and began receiving text messages on their personal cell phones the day after discharge. Text messages were composed to reflect literature published for patient education by the American Heart Association [20]. In addition, language similar to that used by the UCMC inpatient heart failure education team was incorporated into the texts. For a 30-day period, each participant received automated messages in the following domains:

1. Medication adherence: a daily reminder message (eg, “Time to take your heart failure medications”) and a biweekly adherence question (eg, “Did you take all your heart failure medications today?”)

2. Dietary compliance: educational messages (eg, “Remember to avoid salt. Items high in salt include canned soups, deli meats, and fried foods.”)

3. Appointment adherence: a reminder 48 hours before and the day of their cardiology or primary care follow-up appointments (eg, “Please remember to go to your appointment with Dr. Smith today. Take all your medicines with you.”)

4. Heart failure signs and symptom recognition: warning signs of heart failure (eg, “Know the signs of fluid buildup: your weight going up, swelling of your legs, and having trouble breathing.”)

5. Management if experiencing symptoms (“Have you noticed that your legs are swollen or are you having trouble fitting into your shoes? If yes, call your physician.”)

6. Health care navigation: knowing how to get in touch with cardiologist, obtaining medications after discharge (“If you have not done so already, make sure you have all the medicines that you were discharged on.”), and dealing with complications of paying for medications (“If you’re having trouble paying for your medicines, please make sure your doctor knows about this.”)

Each participant’s text message programming was personalized to reflect his/her medication regimen and follow-up appointments. Participants were provided a tutorial on receiving, reading, and sending text messages on enrollment. At the time of enrollment, each patient was sent a test message and replied to it, ensuring basic competency with the use of text messages. Participants were regularly reminded that the system was automated and was not an emergency response system.

### Data Analysis

Per the most recent scoring procedure [18], raw scores from the SCHFI were tabulated into standardized 100-point scales: maintenance, management, and confidence. Preintervention and postintervention scores for each scale were compared using paired *t* tests. Individual items were compared using Wilcoxon rank sum tests. Stata version 11 was used for the analysis (StataCorp LP, College Station, TX, USA).

## Results

### Study Recruitment and Sample Characteristics

Of 61 patients initially identified for ADHF, 51 were successfully approached for enrollment prior to discharge and 27 agreed to participate. Twelve of the patients approached did not own a personal cell phone. An additional 6 patients did not meet inclusion criteria because they failed their Mini-Mental exam (n=3), needed a health care proxy (n=2), or were hard of hearing (n=1). Only 3 patients approached for the study who met all inclusion criteria refused to participate. Of the 27 patients who met inclusion criteria, 15 were successfully enrolled. The remainder were unable to be enrolled due to logistical barriers (eg, off the floor, discharged early). Eight of 15 enrollees completed the text messaging portion of the study. Of the remaining 7 participants, 2 died, 1 was admitted to a subacute facility, and 4 had technology issues, including their cell phone being disconnected. A total of 6 participants completed the entire study including preintervention and postintervention surveys.

All but 1 participant in the study was African American (Table 1). The average age of participants was 50 years (range 23-69) and 40% (6/15) were female. The majority had Medicaid as primary or secondary insurance with Medicare or were uninsured. Approximately half of participants (47%, 7/15) had systolic heart failure. The mean ejection fraction for those with systolic dysfunction was 22%, and two-thirds (67%, 10/15) of all participants had at least 1 prior hospitalization in the past year. Most participants were on evidence-based heart failure therapies on admission including angiotensin-converting enzyme (ACE) inhibitors (53%, 8/15) and beta-blockers (86%, 13/15).

### Cellular Phone Use

Most participants (93%, 14/15) carried their cell phone with them always or almost always (Table 2). All participants reported being somewhat or very comfortable with text messaging, although actual usage varied widely from 0 to 60 text messages per day. All but 1 participant had an unlimited text messaging plan, and only one-third (33%, 5/15) of participants in our sample had a smartphone capable of accessing the Internet and running applications (apps).

**Table 1:** Participant characteristics at enrollment (N=15).

Baseline characteristic		Statistic
Age, mean (range)		50 (23-69)
**Race, n (%)**		
	African American	14 (93)
	White	1 (7)
**Gender, n (%)**		
	Women	6 (40)
	Men	9 (60)
Mini-Mental status, mean (range)		21 (18-22)
**Insurance status, n (%)**		
	Medicare only	3 (20)
	Medicaid only	3 (20)
	Dual eligible	4 (27)
	Private insurance	3 (20)
	Uninsured	2 (13)
**Medical history**		
	Preserved ejection fraction (EF) heart failure, n (%)	8 (53)
	Average EF for those with systolic heart failure (%), mean (range)	22.4 (9-47)
	Hypertension, n (%)	11 (73)
	Diabetes, n (%)	8 (53)
	Smoker, n (%)	5 (33)
	ACE inhibitor on admission, n (%)	8 (53)
	Beta-blocker on admission, n (%)	13 (86)
**Admissions**		
	1 admission in the prior year, n (%)	6 (40)
	2 or more admissions in the prior year, n (%)	4 (27)
	Number of admissions in the prior year, median (range)	1 (0-7)

**Table 2:** Prior participant experience with cellular phone calling and text messaging (N=15).

Baseline characteristic		n (%)
Owns a smartphone		5 (33)
Unlimited text messaging		14 (93)
Landline in addition to cell phone		5 (33)
**Frequency with which carry cell phone**		
	Always	12 (80)
	Almost always	2 (13)
	Sometimes	1 (6)
**Comfort level making or receiving calls**		
	Very comfortable	14 (93)
	Somewhat comfortable	1 (6)
**Total calls made/received per day**		
	0	0 (0)
	1-5	3 (20)
	6-10	3 (20)
	11-20	4 (27)
	>20	5 (33)
Have used text messaging feature before		15 (100)
**Comfort level using text messages**		
	Very comfortable	10 (66)
	Somewhat comfortable	5 (33)
**Total text messages sent/received per day**		
	0	1 (6)
	1-5	4 (27)
	6-10	5 (33)
	11-20	1 (6)
	>20	4 (27)

### Participant Engagement

Although not required, participants were encouraged to text back comments or responses to questions sent via text message. Although responses were not read by research staff during the course of the study, response rate was considered to be a marker of patient engagement and could inform future program design. Over the 30-day intervention, participants sent an average of 5.7 text messages (range 0-27) or approximately 1 message every 5 days. Five participants did not send any text messages; 2 participants sent over 20 messages. Interestingly, both of these participants were near the median in terms of prior usage of text messaging, and 1 reported being only somewhat comfortable with text messaging prior to the study.

### Participant Experience

All (100%, 6/6) participants reported the highest level of satisfaction with the mobile phone–based heart failure self-management program (Table 3). Although most participants (66%, 4/6) strongly agreed that the text messaging system was easy to use and was helpful in improving self-management, a minority (33%, 2/6) strongly disagreed with these statements. Despite this, all participants agreed that they would recommend the program to a friend or family member.

**Table 3:** Participant evaluation postintervention (n=6).

Survey question	Likert scale response, n (%)			
	Strongly agree	Moderately or slightly agree	Slightly or moderately disagree	Strongly disagree
Overall, I was satisfied with this study	6 (100)			
It was easy to receive and read the text messages from the research team	5 (83)			1 (17)
It was easy to send text messages to the research team	4 (66)			2 (33)
I found the text message reminders to be helpful at decreasing the number of pills I missed	4 (66)	1 (17)		1 (17)
I found the text message reminders to be helpful at decreasing the amount of salt in my diet	4 (66)	1 (17)		1 (17)
I found the text message reminders to be helpful at decreasing the number of doctor visits that I missed	3 (50)	1 (17)		2 (33)
I would recommend this cell phone reminder system to my friends/family that have heart failure	5 (83)	1 (17)		

During the open-ended survey, participants reported that the intervention improved self-management directly by providing reminders, but also indirectly by increasing disease awareness and reinforcing the importance of self-management. They liked that the system served as a reminder and provided feedback. One participant stated, “I knew I wasn’t being forgotten,” and another that, “It’s nice to know that someone cares.” Only 2 participants identified elements they did not like about the system: 1 complained that it was hard for him to text and the other wanted more text messages. Participants suggested improvements such as providing more instruction on how to text with the system and making the cost of texting with the system free.

## Change in Heart Failure Self-Management

Participants’ responses to the SCHFI suggested that the intervention was associated with improvements in heart failure self-management (Table 4). On a 100-point standardized scale, self-care maintenance improved from 49 to 78, representing an increase of 28 points (95% CI 15-42, *P*=.003). Self-care management increased from 57 to 86, or 30 points (95% CI 17-42, *P*=.002). There was no statistically significant change in self-care confidence (57 to 75, 95% CI –6 to 43, *P*=.11).

Of the 22 individual items comprising the SCHFI, improvements were seen in 6 items: weighing self, eating a low salt diet, forgetting to take medicines, avoiding getting sick, contacting physician in case of worsening symptoms, and confidence in evaluating symptoms (Table 5). The text messaging intervention specifically targeted content areas covered by 7 of the 22 SCHFI items. For example, the intervention did not include any messages about exercising or using pill counters. Of the 6 individual SCHFI measures that improved, 5 were specifically targeted by the texting intervention. Of the 7 items targeted by the intervention, 5 improved and 2 had no statistically significant change.

**Table 4:** Self-Care of Heart Failure Index (SCHFI) scales preintervention and postintervention (n=6).

SCHFI scale	Preintervention score, mean (95% CI)	Postintervention score, mean (95% CI)	Difference, mean (95% CI)	*P* value
Maintenance	49 (38-61)	78 (68-88)	28 (15-42)	.003
Management	57 (42-71)	86 (72-100)	30 (17-42)	.002
Confidence	57 (32-81)	75 (70-80)	19 (–6 to 43)	.11

**Table 5:** Self-care Heart Failure Index (SCHFI) individual items preintervention and postintervention (n=6).

SCHFI Item		Preintervention		Postintervention		*P* value^a^
		Mean	SE	Mean	SE	
**Maintenance**						
	Weighing self^b^	1.6	0.27	3.3	0.21	*.03*
	Eating low salt diet^b^	2.4	0.27	3.7	0.21	*.03*
	Forgetting to take medicines (lower is better)^b^	1.9	0.22	1.3	0.21	*.02*
	Keeping to low salt diet when eating out	1.5	0.22	3	0.52	.05
	Checking ankles for swelling^b^	3.3	0.25	3.8	0.17	.16
	Keeping appointments^b^	3.3	0.23	3.7	0.33	.45
	Avoiding getting sick	2.9	0.27	3.8	0.17	*.046*
	Physical activity	2.4	0.24	3.2	0.4	.16
	Exercising for thirty minutes	1.3	0.15	2.5	0.56	.11
	Using a pill system	3.1	0.34	2.7	0.61	.51
**Management**						
	Likelihood of contacting physician^b^	2	0.3	3	0.37	*.02*
	Realization of symptoms	2.4	0.31	4	0	.09
	Likelihood of reduction in salt intake	3	0.3	4	0	.16
	Likelihood of reduction in fluid intake	2.8	0.28	4	0	.08
	Likelihood of taking extra diuretics	2.8	0.28	3	0.63	.28
	Thought that remedy helped symptoms	1.9	0.31	3.3	0.33	.11
**Confidence**						
	Confidence in evaluating symptoms^b^	2.8	0.22	3.3	0.21	*.046*
	Confidence in remaining free of heart failure	2.4	0.13	3	0.37	.16
	Confidence in following treatment advice	2.7	0.23	3.5	0.22	.10
	Confidence in recognizing changes	3.1	0.27	3.7	0.21	.23
	Confidence in action to relieve symptoms	2.6	0.24	3.2	0.4	.52
	Confidence in evaluation of remedy	2.6	0.24	2.8	0.31	.73

^a^ Statistically significant values in italics.

^b^ Relates to specific text messages.

There were no statistically significant differences in preintervention SCHFI measures between the 6 patients who completed the entire intervention and the 9 patients who did not complete the intervention or who were lost to follow-up.

## Discussion

We report the results of a text message–based self-management intervention in patients discharged from the hospital with acute decompensated heart failure (ADHF). To the best of our knowledge, this is the first study of a text messaging intervention for ADHF piloted in an urban, largely African American population.

### Principal Results

The text messaging intervention was associated with improvements in self-care maintenance and management. Guidance from the developers of the SCHFI suggests that an improvement in either of these scales of 0.5 standard deviations or 8 points is clinically relevant and that a cutoff score of 70 can be used to judge self-care adequacy [18]. We observed increases in maintenance and management of 28 and 30 points, respectively, suggesting that our findings are clinically significant. Moreover, none of participants scored below 70 preintervention for maintenance, whereas all but 1 improved to above 70 at follow-up; for management, only 1 participant scored above 70 preintervention, but following the intervention all participants scored above the 70 threshold.

Without a control, these results must be interpreted with caution. In addition to a Hawthorne effect, participants were discharged from the hospital and likely seen in clinic at least once or twice between the preintervention and postintervention periods. Self-management teaching at any of these time points, or simply improvement in health status, may account for the observed improvements. However, it is notable that the specific SCHFI items that improved were generally those targeted by the texting intervention, implicating a causal link. Future studies should validate these preliminary findings in a controlled trial.

A major aim of this study was to access the feasibility of text messaging interventions in our patient population. Among patients who met the inclusion criteria, 90% agreed to participate in the study, which is high for our institution. Most participants in our study were comfortable with text messaging and had unlimited text messaging plans. This suggests that in our study population, among those with access to a cell phone, text messaging is a familiar and acceptable means of health care communication. In contrast, only one-third of patients had smartphones, suggesting that mobile phone–based interventions requiring Web access or apps would have low feasibility in our study population.

However, 24% of patients approached for recruitment did not own a personal cell phone. This differs from national surveys in which high rates of mobile phone access in low-income populations are observed [12]. However, these surveys were conducted in the general population, not hospitalized patients with complex medical needs. Future studies in low-income populations should consider providing participants cell phones to improve accessibility.

There were considerable challenges to texting in our study. Although all participants reported comfort in texting prior to enrollment, we observed low participant response rates and requests for additional training in texting. Although most participants reported high levels of satisfaction with the system, a few found the system difficult to use and not helpful in improving self-management. More research is needed in how to design technologies that are usable across a wide range of patients and how to best target mobile phone–based interventions to patients most likely to benefit.

### Limitations

A major limitation of our study was the rate of completion and loss to follow-up. Of the 15 participants enrolled, only 6 received the entire intervention and completed preintervention and postintervention surveys. Although this largely reflects the challenges with research in our study population, it may bias the results toward those who responded favorably to the system. The study also had no control group, so the effects on self-management should be interpreted with caution. Finally, this was a single institution study with a small sample size and the results may not generalize to other patient populations.

### Comparison With Prior Work

Prior mobile phone-based heart failure interventions have had mixed results when studied in clinical trials [14,15]. In contrast to our intervention, which focused on providing self-management support, these studies used mobile phones largely as telemonitoring devices. The Telemonitoring to Improve Heart Failure Outcomes (Tele-HF) study, a large negative multicenter clinical trial, demonstrated that telemonitoring is not effective in reducing death or hospitalization in patients recently admitted for ADHF [21]. In our study, rather than facilitating in-between visit care by providers, we aimed at building self-care skills through reminders, encouragement, and patient education. Our study builds on prior qualitative work suggesting that the effects of mobile phone-based interventions in heart failure may go beyond telemonitoring and increase patient empowerment [22], and a recent clinical trial suggesting that self-management education is particularly effective in low-income patients with heart failure [16]. It also extends findings from other chronic diseases, including diabetes, asthma, and human immunodeficiency virus/acquired immunodeficiency syndrome, on the utility of text message-based interventions in urban African Americans [19,23-25].

### Conclusions

Text messaging may be a useful tool for improving heart failure self-management in urban African Americans after hospital discharge. More research is needed to target enrollment to those patients most likely to benefit and to evaluate outcomes in a controlled trial.

## Abbreviations

ADHF: acute decompensated heart failure
EF: ejection fraction
SCHFI: Self-Care of Heart Failure Index
UCMC: University of Chicago Medical Center
